# Daratumumab, Lenalidomide, and Dexamethasone (DRD), an Active Regimen in the Treatment of Immunosuppression-Associated Plasmablastic Lymphoma (PBL) in the Setting of Gorham's Lymphangiomatosis: Review of the Literature

**DOI:** 10.1155/2022/8331766

**Published:** 2022-06-27

**Authors:** Matthew Lee, Beth A. Martin, Haifaa Abdulhaq

**Affiliations:** ^1^UCSF Fresno, Department of Internal Medicine, Fresno, CA, USA; ^2^Stanford University, Division of Hematology, Stanford, CA, USA; ^3^UCSF Fresno, Division of Hematology and Oncology, Fresno, CA, USA

## Abstract

Characterized by an aggressive course with a poor overall survival due to treatment refractoriness, plasmablastic lymphoma (PBL) is a rare variant of diffuse large cell B cell lymphoma. Gorham's lymphangiomatosis or Gorham–Stout disease (GSD) is a rare skeletal condition of unknown etiology characterized by progressive bone loss and nonmalignant proliferation of vascular and lymphatic channels within the affected bone. Neither disease has a standard of care. We present a 23-year-old HIV-negative woman with GSD, managed medically with octreotide and sirolimus, who developed PBL. After progressing on V-EPOCH (bortezomib, etoposide, vincristine, cyclophosphamide, doxorubicin, and prednisone), she was treated with daratumumab, lenalidomide, and dexamethasone (DRD) therapy and achieved complete remission after two cycles with progression after eight cycles. This is a report of treatment of PBL with DRD therapy. Clinical investigations of the DRD regimen in PBL in conjunction with other agents to improve both depth and durability of response are warranted.

## 1. Introduction

Plasmablastic lymphoma (PBL) is highly associated with a compromised immune system, with the majority of the cases found in HIV-positive individuals. HIV-negative disease occurs with a slight male predominance (male-to-female ratio of 1.7), a median age of 55 years, oral mucosal tropism, and is associated with a worse overall median survival [1–3]. Among this population, apparent immunocompetence exists in 80%, with the remainder having a concurrent lymphoproliferative or autoimmune disorder. Approximately 10% developed PBL after receiving a solid organ transplant [4, 5]. Importantly, Epstein–Barr virus (EBV) positivity is found in approximately half of HIV-negative cases. Both HIV and EBV-negative diseases are also associated with a decreased event-free survival [2, 3].

Plasmacytic differentiation, CD38+, high MYC expression secondary to translocation (8; 14), high Ki-67 activity, positive MUM-1, and absence of B cell markers such as CD20 are the key features of the histology of PBL [4, 6]. Distinguishing PBL from plasmablastic myeloma is difficult [7, 8]. PDL1 staining is positive in 25% of tumor cells, and PD1 staining is positive in the majority of infiltrating cells [2]. Currently, there is no standard treatment regimen for PBL. Dose-adjusted EPOCH or V-EPOCH is often utilized [9].

While the exact mechanism of GSD development is unknown, lymphangiogenic growth factors may be drivers [10, 11]. Although the majority of patients are younger than 40 years of age, no genetic predisposition has been found. GSD occurs in both genders and at all ages. Any bone can be involved with the most common sites being the hip and shoulders. The rarity of the disease and the limitation of treatment data to case series confine the standard of care to supportive surgical intervention and medical management. Medical therapy primarily utilizes inhibitors of lymphatic endothelial cell markers, such as sirolimus.

We present a case of PBL associated with sirolimus in a patient with GSD who achieved a complete response to treatment with daratumumab, lenalidomide, and dexamethasone.

### 1.1. Case Presentation

The patient was a 23-year-old Hispanic female in whom GSD was diagnosed at age 5 when she was found to have an absent left clavicle. Her manifestations of GSD were lymphedema of left upper extremity, resorption of the left clavicle, scapular head and humerus, chylothoraxes requiring chest tubes, and recurrent pericardial effusions requiring a window. Treatment with sirolimus and octreotide, for nearly 3 years, controlled lymphedema and effusions.

**Figure 1:**
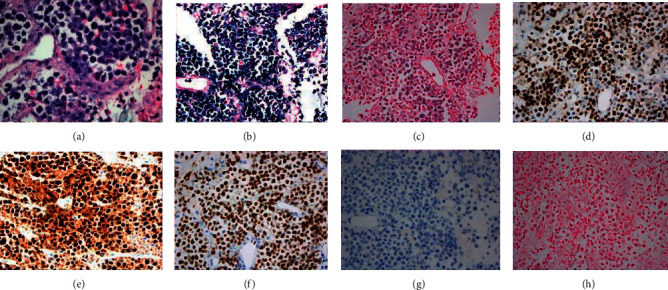
Pathology slides. (a) H&E stain of core biopsy. (b) High Kappa expression. (c) Negative lambda expression. (d) Positive cMYC expression. (e) Positive MUM-1 expression. (f) High Ki-67 expression. (g) Negative CD20 expression. (h) Negative EBER ISH.

She developed pneumonia and hypoxic respiratory failure which required intubation. No oropharyngeal lesions were detected. CT scan of the chest, abdomen, and pelvis showed markedly enlarged supraclavicular, mediastinal, left axillary, and thoracic wall conglomerate, as well as enlargement of the thyroid with a significant mass effect on the trachea. Due to critical illness, biopsy was limited to a fine needle aspiration (FNA) of a left axillary lymph node and revealed sheets of cells with plasma cast and revealed sheets of cells with plasmablastic morphology. Immunohistochemical stains showed that the malignant cells were kappa restricted with MYC overexpression (>80%) and MUM-1 and CD 138 expression, Ki-67 > 90%. The cells were negative for CD20, CD30, CD3, ALK, BCL6, BCL2, and HHV8. Notably, EBV in situ hybridization was negative (Figure 1). PD1 and PDL1 stainings were not performed. By serology and PCR on blood, HIV, EBV, and human herpes virus 8 (HHV8) were negative.

Flow cytometry from FNA revealed an abnormal population of CD45-negative events, expressing bright CD38 and partial dim CD20. No definite surface light chain expression was detected. CD19, CD5, CD10, and any T cell antigens were not coexpressed.

Fluorescence in situ hybridization (FISH) was positive for MYC gene rearrangement t(8; 14) and negative for both BCL2 and BCL6 translocation.

**Figure 2:**
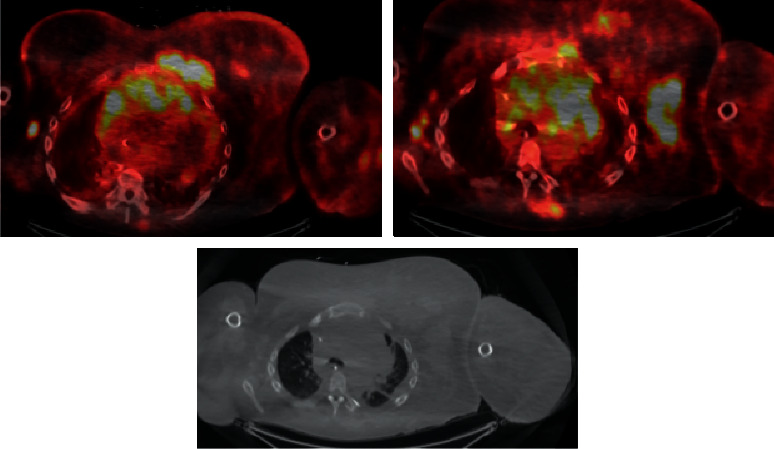
Initial PET/CT showing significant hypermetabolic activity above the diaphragm specifically located in the L axillary, mediastinal, and thoracic wall conglomerate.

PET/CT revealed hypermetabolic lymphadenopathy above and below the diaphragm, extensive conglomerate mass of the chest wall, left upper extremity, mediastinum, and left breast, and hypermetabolic involvement of the thyroid gland bilaterally with narrowing of the trachea. No bone lesions were found. SUV ranged from 4 to 12.8 with mean 9.4 and median 10.1 (Figure 2). Given the overall features of plasmablastic morphology and immunophenotype, nodal involvement without bone lesions, PBL was favored over plasmablastic myeloma.

Sirolimus was discontinued. Dose-adjusted V-EPOCH was initiated and was complicated by neutropenic fever despite high-dose granulocyte colony stimulating factor support. Cycle 2 of V-EPOCH was complicated by small bowel obstruction, prolonged neutropenia, and slow functional recovery. Repeat CT of the chest, abdomen, and pelvis revealed progression of disease in the small bowel and peritoneum. Treatment with lenalidomide, dexamethasone, and daratumumab as per the DRD regimen was initiated [12].

**Figure 3:**
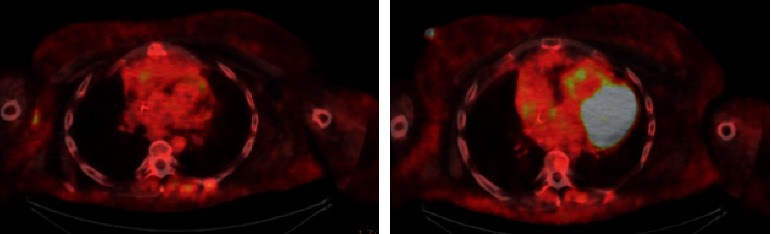
PET/CT after 2 rounds of DRD therapy showing significant improvement in hypermetabolic activity seen in previous imaging consistent with complete response (Deauville 2).

Repeat PET-CT after 2 cycles of DRD showed significant improvement in the size of the nodes and a metabolic complete response in all nodes (Deauville 2) (Figure 3). In addition, the DRD regimen controlled her GSD-associated lymphedema and effusions.

Autologous stem cell transplantation (ASCT) was considered. After 8 monthly cycles of DRD, PBL progressed. No IVIG was given. Standard dose carfilzomib was added to the regimen for one cycle without improvement. ICE (ifosfamide, carboplatin, and etoposide) regimen was initiated without improvement. She expired 13 months after diagnosis.

## 2. Discussion

This patient had two diseases that required active management: GSD and HIV-negative and EBV-negative immunosuppression-associated PBL. As stated above, both HIV-negative and EBV-negative statuses have been independently linked to worse overall survival [2, 3].

Plasmablastic lymphoma is a rare disease which makes up approximately 2% of HIV-associated lymphomas. The incidence of the disease is unknown, and it has been reported in patients of all ages.

Review of the database from eighteen Surveillance, Epidemiology, and End Results (SEER) registries showed median age at diagnosis was 52 years with male predominance (81.9%) and majority of patients were non-Hispanics (76.6%). Patients were slightly more frequently diagnosed with advanced stage (III-IV) (59.0%). Extranodal presentation was more common than nodal (54.8% vs. 45.2%). Oral and gastrointestinal (GI) sites were the most frequent primary extranodal locations (23% and 19.4%, respectively). Oral primary location was inversely associated with the presence of B symptoms and advanced Ann Arbor stage [13].

PBL in HIV-negative patients is more heterogeneous in sites of involvement with advanced clinical stage, B symptoms, and bone marrow involvement being less common (25%) than in HIV-positive patients.

The specific pathogenesis of PBL is not well understood, and the cell of origin is thought to be the plasmablast, an activated B cell that has undergone somatic hypermutation and class switching recombination and is in the process of becoming a plasma cell. Recent studies have identified the presence of *MYC* gene rearrangements in addition to the association with EBV infection as important pathogenic mechanisms [4].

EBV infection is associated with prevention of apoptosis in B cells by several mechanisms related to EBV antigens. *MYC* dysregulation mediated by translocation or amplification may cause plasmablast development and prevent further differentiation. *MYC* translocations may allow PBL cells to escape apoptosis [4].

Finally, recurrent mutations in PRDM1, a gene that encodes the plasma cell transcription factor Blimp1 protein, have been described. These mutations in PRDM1 could alter the regulation of different targets, including *MYC*, in these lymphomas [13].

GSD is an extremely rare condition with no current consensus on management, as options are limited to case reports. One of the complications of the disease is the development of a chylothorax, which has a reported mortality rate of as high as 64% with medical management and 36% with surgical treatment [14, 15], and can result in significant morbidity if chronic drainage is required. Thalidomide is a known treatment for GSD complicated by the chylothorax and for multiple myeloma, thus making imides, including lenalidomide, a rational option [16–18].

PBL is a difficult condition to treat with a poor overall survival of 6–19 months and without standard treatment regimens [3, 19–21].

Improved survival was reported in PBL patients treated with chemotherapy recently. The 3-year and 5-year overall survival (OS) rates of treated PBL patients in SEER registries were 54% and 52.8%. HIV status did not affect survival outcomes in unadjusted and adjusted analyses [22].

In a study of 135 PBL patients reported by the Lymphoma Study Association (LYSA), 80% of patients received chemotherapy and their median OS was 32 months [23].

V-EPOCH has been associated with high complete response (CR) rates and improved overall survival [2, 19]. In a case series by Dittus et al., there was 100% complete response rate and 2-year overall survival of 50% with V-EPOCH therapy [24]. ASCT and, more recently, chimeric antigen-receptor T cell therapy (CAR-T) are considered for consolidation [25, 26]. Single agent lenalidomide has been effective in case reports of PBL throughout the literature, most recently by Ando et al. [27–29]. A recent review of 173 patients with PBL supports a wide range of therapies. None received daratumumab [30].

Surface signaling molecule expression can yield opportunities for utilization of antibody or cell-based therapeutics: CD19 (CAR-T), CD38 (daratumumab, isatuximab, and CAR-T directed to CD38 or CD138), CD 30 (brentuximab vedotin, and CAR-T directed to CD30), and PD1/PDL1/PDL2 (checkpoint inhibitors) [26].

The strong CD38+ expression that could be responsive to daratumumab, as well as the potential for dual activity of lenalidomide in PBL and GSD, supported the use of the DRD regimen in this patient.

To our knowledge, there are no reported cases of attaining a complete response of PBL with DRD therapy. Eight cases of use of daratumumab in the treatment of plasmablastic neoplasms have been published. In one HIV-negative patient with plasmablastic myeloma, three cycles of daratumumab and CHOP (cyclophosphamide, doxorubicin, vincristine, and prednisone) resulted in a partial response [31]. The second patient, who had HIV-positive PBL, progressed after 8 cycles of EPOCH and remitted after 3 cycles of combination of dexamethasone, cisplatin, cytarabine (DHAP regimen), bortezomib, and daratumumab. ASCT and maintenance lenalidomide resulted in a sustained remission [32]. The third case presents a HIV-negative patient who developed PBL from chronic lymphocytic leukemia transformation. The patient underwent therapy with daratumumab and CHOP with only a transient response, thus unable to proceed to allogenic stem cell transplant (alloSCT) [33]. The fourth case presents a female with HIV-negative disease who received multiple lines of treatment (daEPOCH, ASCT, and DHAP) before achieving CR with bortezomib, lenalidomide, and daratumumab and eventually proceeding to alloSCT with a prolonged disease-free period [34]. Raychaudhuri et al. were unable to induce remission with salvage carfilzomib and DRD in a HIV-negative and EBV-negative patient, eventually proceeding to CAR-T therapy [25]. Roche et al. found that daratumumab as a single agent was an ineffective salvage therapy in 3 patients with PBL [35]. A clinical trial of daratumumab with dose-adjusted EPOCH is underway for HIV-associated and non-HIV-associated PBL by the AIDS Malignancy Consortium [36]. Ricker et al. described a case series of four patients with advanced PBL who received daratumumab in combination with chemotherapy. All four subjects had CR and three had a durable response of at least 8 months (range 8–17 months) [37].  Table 1 provides a summary of cases of PBL treated with daratumumab.

CD30 is a viable target for PBL as positive expression has been found in approximately 30–50% of PBL cases [8]. Two case reports of brentuximab vedotin use both leading to impressive tumor reduction but also fatal outcome due to tumor lysis syndrome and comorbidities [38, 39]. There are currently multiple trials investigating the use of EBV cytotoxic lymphocytes expressing CD30 chimeric receptors in the use of CD30+ Hodgkin's and non-Hodgkin's lymphomas (e.g., NCT01192464 and NCT02917083), with Ramos et al. showing durable responses in patients with refractory NHL [40].

In a study by Laurent et al. on 82 patients with PBL, nearly all cases expressed PDL1 and PD1 in the immune infiltrate, and one-quarter of them strongly expressed PDL1 in tumor cells and in immune cells. PD1/PDL1 were more overexpressed in the microenvironment in EBV^+^PBL [2]. As a result, immunotherapy is emerging as a treatment option for this disease. In a report of salvage therapy with single agent nivolumab in a patient with PDL1+ PBL, treatment was used as a successful bridge to allogeneic stem cell transplant [41]. In another report of chemoresistant EBV + PBL, one patient treated with tislelizumab and lenalidomide, the treatment resulted in CR with total overall survival of 18 months [42].

CD19 CAR-T therapy was described in a case report about a patient with relapsed PBL who had complete response with axicabtagene ciloleucel at 4 months with an undetectable CD-19+ count 2 months after administration. Unfortunately, the patient relapsed at 5 months with loss of CAR T cell activity [25].

Stem cell transplantation in PBL has been assessed as either consolidation in chemotherapy sensitive disease or as salvage therapy [43]. There are multiple case series reporting favorable response to ASCT. Liu et al. reported 4 cases of HIV-negative PBL undergoing ASCT in CR1 with sustained remission [3]. Cattaneo et al. treated 24 patients with ASCT with 7 relapsing at 2 years [44]. Although the data are extremely limited, long-term survivorship has been reported in alloSCT for PBL. Hamadani and Devine achieved CR in a HIV + PBL patient [45]. As reported earlier by Kathrotiya et al., the patient has been disease-free for almost 3 years after undergoing haploidentical alloSCT [34]. Rong et al. achieved complete remission in a HIV-negative and EBV-negative patient for 4 years after salvage alloSCT [46].

## 3. Conclusion

Based on this patient's experience, the DRD regimen has a significant activity in treatment of both PBL and GSD. The response and drug mechanisms suggest noncross resistance. We speculate that maximizing immunocompetency as soon as possible, e.g., by minimizing intensive corticosteroids and myelosuppressive chemotherapies after initial deep remission, may be a beneficial strategy. Furthermore, given that the DRD regimen is not readily available as an emergency intervention in the inpatient setting, we anticipate that an EPOCH-based regimen will remain as the preferred upfront induction regimen. We believe that testing DRD regimen in conjunction with other therapeutics such as checkpoint inhibitors, cellular therapeutics such as CAR-T, and/or stem cell transplantation with curative intent in PBL is warranted.

## Figures and Tables

**Table 1:** Summary of reported uses of daratumumab in PBL.

Reference	HIV status	EBV status	Stage	Lines of tx before Dara	Prior SCT	Dara regimen	Outcome
Suarez et al. [31]	—	—	IV	0	n/a	Dara + CHOP	PD
Chikeka et al. [32]	+	+	IV	1	n/a	Dara + V + DHAP	CR-ASCT
Marvyin et al. [33]	—	n/a		0		Dara + CHOP	PD
Kathrotiya et al. [34]	—	+	IV	2	ASCT	Dara + VR	CR-AlloSCT
Raychaudhari et al. [25]	—	—	IV	2	n/a	DRD + carfilzomib	PD-CAR-T
Roché et al. [35]	—	—	IV	4	ASCT	Dara alone	PD
Roché et al. [35]	—	+	II	2	n/a	Dara alone	PD
Roché et al. [35]	—	—	II	1	ASCT	Dara alone	PD
Ricker et al. [37]	—	+	IV	0	n/a	Dara + EPOCH	CR
Ricker et al. [37]	+	+	IV	0	n/a	Dara + EPOCH	CR
Ricker et al. [37]	—	+	IV	0	n/a	Dara + EPOCH	CR
Ricker et al. [37]	—	—	IV	0	n/a	Dara + EPOCH	PD

CHOP, cyclophosphamide, hydroxydaunorubicin, vincristine, and prednisone; V, bortezomib; DHAP, dexamethasone, cytarabine, and cisplatin; VR, bortezomib, rituximab; PD, progressive disease; CR, complete response.

## Data Availability

The data used to support the findings of this study are available from previous medical studies and case reports which are reported in the references section found on MedHub.
